# Do Single Men Smell and Look Different to Partnered Men?

**DOI:** 10.3389/fpsyg.2019.00261

**Published:** 2019-02-13

**Authors:** Mehmet K. Mahmut, Richard J. Stevenson

**Affiliations:** Food, Flavor and Fragrance Lab, Department of Psychology, Macquarie University, Sydney, NSW, Australia

**Keywords:** mate preferences, mate attraction, masculinity, body odor, face attractiveness

## Abstract

Previous research indicates human body odor (BO) can signal kinship, sickness and genetic compatibility. Based on research indicating single males have higher testosterone levels than partnered males and that higher testosterone levels are associated with stronger smelling BO, the current study aimed to determine if, by extension of previous findings, single males’ BO smells stronger than partnered males’ BO. Eighty-two heterosexual women aged 18–35 years rated the BO and faces of six different males also aged 18–35 years. Consistent with the hypothesis, single men’s BO smelled stronger than partnered men’s BO and single men’s faces were rated as more masculine than partnered men’s faces. The possible advantages of females being able to identify single males are addressed in the Discussion.

## Introduction

Humans rely heavily on visual cues to make mate preference judgements. From an evolutionary perspective, mate preferences based on facial attractiveness is advantageous for identifying and selecting a high quality partner (Buss and Schmidt, 1993). For example, research findings have demonstrated that facial attractiveness (Coetzee et al., 2009) and color (Stephen et al., 2011) are associated with physiological health. However, despite the vast majority of research focussing on signals detected by the visual sense, humans do not rely solely on visual cues to assess the suitability of a potential partner but also make judgements using their sense of smell (Stevenson, 2009). Specifically, the body odor (BO) of a potential partner is assessed by our sense of smell (Lübke and Pause, 2015) and given BOs can signal physical health and genetic compatibility with a potential partner, the role of BOs in mate attraction, and preference is not surprising.

In terms of our health, some infections (e.g., gangrene), and diseases (e.g., diabetic ketoacidosis) cause our bodies to emit odors that physicians can reliably recognize and use for diagnostic confirmation (Bijland et al., 2013). In terms of the genetic compatibility of a couple, a set of genes encoding the major histocompatibility complex (MHC) – cell-surface proteins involved in pathogen resistance (Milinski, 2006) that influence our BO (Milinski et al., 2013) – may also contribute to mate preference based on BO preference. For example, women have demonstrated a preference for the BO of men who have dissimilar MHC (Wedekind et al., 1996; Wedekind and Füri, 1997; Sorokowska et al., 2018) and offspring from MHC dissimilar (vs. similar) parents are potentially healthier. However, a recent meta-analysis (Winternitz et al., 2017) on the role of MHC in mate preference in various studies (not just those on BO-based preferences), concluded that mate choice was not driven by MHC differences.

Human BOs are not static and can change due to many factors, such as diet and menstrual cycle. For example, a study that experimentally controlled the amount of red meat consumed over a two-week period, found that a diet higher in meat is associated with unpleasant smelling BO compared to a non-meat diet (Havlíček and Lenochova, 2006). However, it must be noted that Zuniga et al. (2017) found that higher meat consumption was associated with more pleasant smelling BO, although meat consumption frequency was based on self-report data which may account for the contrary findings to Havlíček and Lenochova (2006). Moreover, men’s preferences for female BO vary based on the different stages of a women’s menstrual cycle which are associated with the most dramatic changes in hormone levels; giving higher preference ratings for women’s BO in the fertile phase of their cycle than those in the non-fertile phase (Gildersleeve et al., 2012).

While research investigating changes in hormone levels predominantly focus on the menstrual cycle, numerous studies have found differences in men’s hormone levels based on their relationship status. Specifically, research findings have shown that heterosexual men with higher levels of testosterone were less likely to be married (Booth and Dabbs, 1993; Mazur and Michalek, 1998; van Anders and Watson, 2007; Van Anders and Goldey, 2010) or in long-term relationships (Gray et al., 2004) whereas lower levels of testosterone were associated with being in a romantic relationship. Further, various hormones (e.g., cortisol and testosterone) may affect the quality of a man’s BO (Rantala et al., 2006) and how attractive they are perceived to be. For example, Thornhill et al. (2013) found that women’s preference for BO of high testosterone men was significantly correlated (*r* = 0.32) with their probability of conception risk, presumably because higher testosterone may confer some form of evolutionary fitness (see Folstad and Karter, 1992). Similarly, Butovskaya et al. (2013) reported that women in the most fertile phase of their menstrual cycle prefer the BO of men with masculine qualities (e.g., social dominance) and numerous studies have shown women prefer BO of men with symmetrical faces (Gangestad and Thornhill, 1998; Thornhill and Gangestad, 1999; Thornhill et al., 2003).

In van Anders and Watson (2006) social neuroendocrinology theoretical framework, they presented evidence detailing the important role testosterone plays in behaviors that predict evolutionary fitness, namely; competition for resources, establishing a pair bond (securing a relationship), sexual activity plus parenting and pregnancy. A prediction arising from this conceptual framework is that higher testosterone levels are associated with competitive behaviors (such as acquiring resources) whereas lower testosterone levels are associated with pair-bond maintenance behaviors (such as intimate contact; van Anders and Watson, 2006). Given the evidence that men’s hormone levels may differ based on their relationship status, and that hormone levels may in turn change the perceptual quality of men’s BO, the aim of the current study was to empirically investigate for the first time whether single and partnered men’s BO was perceptually different. Moreover, to assess the role that both visual and olfactory perception may play in mate preference, two modalities that are predominantly researched independently, the current study also tested whether the faces of single and partnered men differed based on visual ratings.

To determine whether single men’s BO smelled different to the BO of partnered men, heterosexual female participants rated men’s BO on five characteristics (e.g., sexiness, liking). Based on previous research suggesting male testosterone levels were positively (but not significantly) associated with stronger smell BO ratings (Rantala et al., 2006) and single males have higher levels of testosterone (e.g., Booth and Dabbs, 1993), we hypothesized that single men’s BO would smell stronger than that of partnered men’s. Moreover, because stronger smelling BO ratings are associated with lower BO liking ratings (Havlíček and Lenochova, 2006), we predicted that single men’s BO would be liked less and rated less sexy than partnered men’s BO. In order to determine whether BO attractiveness predicted facial attractiveness, participants also rated the faces of the BO donors. Although the findings from three previous studies (Rikowski and Grammer, 1999; Thornhill and Gangestad, 1999; Foster, 2008) indicated the correlation between male BO and face attractiveness ratings made by fertile women is low (e.g., *r* = 0.28, *p* = 0.030; Thornhill and Gangestad, 1999), we hypothesized that favorable BO ratings (i.e., higher liking and sexiness) would be associated with favorable face ratings (e.g., attractive, masculine). We made no *a priori* predictions about differences between single and partnered men’s face attractiveness ratings. Finally, to ensure the ability to compare the BO and face ratings of single and partnered men, participants rated the stimuli of three single and three partnered unknown men.

## Materials and Methods

### Participants

Eight-two (42 single, 40 partnered) heterosexual females (*M* = 20.2 years, *SD* = 2.9) completed the study at Macquarie University for credit towards an introductory psychology course. A single participant was someone who was not in a committed romantic relationship whereas a partnered participant was someone was in a monogamous, romantic relationship. Given single and partnered women may perceive a man’s BO or face differently (e.g., Little et al., 2002) we included both partnered and single women in this study. Participants were asked about their medical history and to indicate whether their sense of smell functioned normally. Only heterosexual females aged between 18 and 35 years, who indicated they had a normal sense of small with no history of a condition, injury or surgery that compromised their sense of smell prior to, or on the day of the study, qualified for the study. Clearance to conduct the study was granted by the Human Research Ethics Committee at Macquarie University’s and all participants and donors gave written and informed consent.

### Donors of Body Odor and Face Pictures

The BOs and face pictures of 91 males formed the stimuli pool for the current study. The donors had no other involvement in the study aside from supplying their BO and face picture. The majority of donors were selected by participants; for partnered participants, the donor was their current partner and for single participants, the donor was their friend or brother. However, the Experimenters also recruited 10 donors to ensure there was a sufficiently large stimulus pool to draw from. All donors had to be aged between 18 and 35 years to qualify for the study. All donors were heterosexual, except for one who identified as homosexual, whose BO was included in the stimulus pool. Overall, 46 of the BO donors were single and 45 were partnered. However, there was no significant difference between single and partnered donors in terms of their Body Mass Index (BMI; 24.8 vs. 24.3) or age (21 vs. 22.5 years).

### Donor Data, Stimuli Collection and Preparation

#### Body Odor Collection and Preparation

Approximately one week before testing, each participant collected a donor pack from the Experimenter. The donor pack included a new, white, 100% cotton T-shirt in a resealable plastic bag, an instructions sheet and short survey containing demographic questions which participants delivered it to their known donor. Odor donors were instructed to avoid eating odorous foods (e.g., garlic, onion; Fialová et al., 2016) 24 h before and while wearing the T-shirt, wash using non-perfumed products before wearing the T-shirt and not to use perfumed products while wearing the T-shirt (Allen et al., 2016). The donor was instructed to wear the T-shirt for one day (i.e., no more than 24 h) and to not remove the shirt until a significant amount of sweat was absorbed onto the underarm of the T-shirt. The instruction sheet included a photograph of a model wearing a white T-shirt depicting an unacceptable amount of underarm sweat (i.e., approximately 25% of underarm patch appeared wet with sweat) and the minimum acceptable amount of underarm sweat (i.e., approximately 75% of underarm patch appeared wet with sweat). The type of physical activity participants engaged in to produce the sweat was not prescribed but it was suggested that brisk walking or sporting activities may facilitate sweating.

After removing the T-shirt, donors were asked to return the T-shirt to the resealable plastic bag provided and immediately store in a freezer. Participants collected the sweated-in T-shirt from donors and brought it in on the day of testing. Participants were informed of the importance of keeping the shirt in a freezer until bringing it into the lab. Upon receiving the T-shirt, the Experimenter cut out both underarms of the T-shirt and placed each in a new separate, opaque, plastic condiment bottle that was approximately 14 cm tall with a 250 mL capacity. Each bottle had a screw-on lid with an elongated nozzle with a removal cap and a 5mm opening through which the odorant was delivered. When not in use, the bottles stored in a freezer, a method validated in previous studies (e.g., Lenochova et al., 2009).

### Face Pictures

Donors also supplied a current, digital, color, passport-style photo (i.e., neutral face, no hat or glasses) which was digitally adjusted using a computer to a height of 8 cm before being printed (in color) on white, A4-sized paper.

### Donor Demographics

Each donors’ height, weight, age, relationship status (i.e., single or partnered) and relationship to participant (i.e., partner, friend, relative) was collected via a short self-report survey that was included in the donor pack.

### Measures

#### Excluded Participants and Variables

Two partnered participants’ data were excluded from analyses because one’s partner was not within the accepted age range (of 18 to 35 years) and the other returned a T-shirt smelling of perfume. Other measures were administered as part of a larger project, namely self-report measures relating to the nature of the donor-target relationship. The results from these measures were unrelated to the aims and hypotheses of the current study and are therefore not reported here. Finally, to remove any bias associated with preference a participant may have for their donor’s BO and/or face, the results presented do not include the data from the ratings participants made of their donor.

### Body Odor and Face Stimuli Selection

The Experimenter selected six different donors’ BO and the six corresponding face pictures which each Participant would be presented in a random order. The first BO and face picture selected belonged to the participant’s donor. The BOs and faces of the next six donors (three single, three partnered) were randomly selected from two separate donor pools; one consisting of single and the other consisting of partnered donors unknown to the participant.

### Body Odor Characteristics Ratings Task

The six BOs were randomly presented to participants who made five ratings of each BO based on the following questions (variable label in brackets): (1) How much do you like/dislike this smell? (“Like”); (2) How sexy does this odor smell? (“Sexy”); (3) How familiar are you with this smell? (“Familiarity”); (4) How strong does this smell? (“Strong”) (5) How much does this odor smell like your odor donor? (“Similarity”), on a 7-point scale from zero (not at all) to six (very). The Experimenter squeezed the bottle containing the BO three times approximately 2.5 cm from participants’ nostrils while participants inhaled through their nose. The minimum inter-stimulus interval was 30-s. For each of the five BO characteristics ratings, two variables were computed: the first was the average rating given by the participant to the BO of partnered donors and the second was the average rating given by the participant to the BO of single donors. Therefore, a total of 10 variables were computed. For example, for the BO “Like” ratings, there were two variables created: one was the BO “Like” rating averaged across all single donors that were rated and the second variable created was the BO “Like” rating averaged across all partnered donors that were rated.

### Face Characteristics Ratings Task

Participants were randomly presented with the six faces corresponding to the six BOs selected and asked to rate each face on eight characteristics that have been found to be universally desired (Buss, 1989, 1994; i.e., Masculine, Good Partner, Sexy, Intelligent, Loyal, Attractive, Kind and Trustworthy) on a scale ranging from zero (not at all) to six (very). For each of the eight face characteristics ratings, two variables were computed: the first was the average rating given by the participant to the faces of partnered donors and the second was the average rating given by the participant to the faces of single donors. Therefore, a total of 16 variables were computed. For example, for the face Masculine ratings, there were two variables created: one was the face Masculine rating averaged across all single donors that were rated and the other was the face Masculine rating averaged across all partnered donors that were rated.

### Procedure

The study was administered by three different female Experimenters, each conducting a similar number of studies.

### Preliminary Data Analysis

Note that we tested whether having a beard influenced face masculinity ratings by comparing face masculinity scores of donors with beards (9% of sample) and without (91% of the sample); the results of an independent samples *t*-tests revealed no significant differences between these groups (all *p*s > 0.05). We also tested, but found no significant differences, between partnered and single female participants or between females using or not using birth contraception in terms of their ratings of single and partnered men’s BO and faces.

## Results

### Were Single and Partnered Men’s BO Rated Differently by Single and Partnered Women?

To determine whether single and partnered female participants rated single and partnered men’s BO differently on five characteristics (i.e., Strong, Like, Sexy, Familiarity and Similarity), five 2 × 2 mixed design analysis of variances (ANOVA) were ran (see Table 1 for descriptive statistics). The between-subjects variable in each ANOVA was Participant Relationship Status (i.e., partnered or single) and the within-subjects variable was Donor Relationship Status which had two levels (i.e., partnered or single). The family-wise error rate was adjusted for the five comparisons made such that the alpha-level was set at 0.01 (i.e., 0.05/5).

**Table 1:** Single and partnered women’s ratings of single and partnered men’s body odor.

	Entire sample (*n* = 82)		Partnered women (*n* = 40)		Single women (*n* = 42)	
	Donor type (Male)		Donor type (Male)		Donor type (Male)	
BO ratings	Single	Partnered	Single	Partnered	Single	Partnered
			
	Mean (SD)	Mean (SD)	Mean (SD)	Mean (SD)	Mean (SD)	Mean (SD)
Strong	3.54 (1.14)	3.04 (1.06)	3.64 (1.12)	3.21 (1.07)	3.43 (1.16)	2.86 (1.05)
Familiarity	1.76 (1.36)	1.76 (1.02)	1.61 (1.42)	1.69 (1.10)	1.93 (1.29)	1.83 (0.94)
Sexy	1.41 (1.20)	1.44 (1.14)	1.37 (1.20)	1.37 (1.14)	1.46 (1.22)	1.51 (1.14)
Like	2.08 (1.27)	2.21 (1.02)	2.04 (1.31)	2.08 (1.07)	2.13 (1.24)	2.34 (0.97)
Similarity	1.61 (1.30)	1.65 (1.08)	1.49 (1.37)	1.76 (1.17)	1.73 (1.22)	1.53 (0.98)

The first ANOVA was conducted with the BO strong ratings as the dependent variable (DV), which revealed a significant main effect for Donor Relationship Status, *F*(1,77) = 9.51, *p* = 0.003, $\eta_{p}^{2}$ = 0.11, indicating that averaged across participants, single men’s BO was rated as smelling stronger than partnered donor’s BO. There was no significant main effect for Participant Relationship Status, *F*(1,77) = 2.16, *p* = 0.15, $\eta_{p}^{2}$ = 0.03, or Participant Relationship Status × Donor Relationship Status interaction (*F* < 1).

The next four ANOVAs revealed no significant main or interaction effects (with 11 of 12 *F*-values < 1) indicating that partnered and single women did not rate partnered and single men’s BO different on BO characteristic ratings of Like, Sexy, Familiarity and Similarity.

### Were Single and Partnered Men’s Faces Rated Differently by Single and Partnered Women?

To determine whether single and partnered female participants rated single and partnered men’s faces differently on eight characteristics (i.e., Masculine, Good Partner, Sexy, Intelligent, Loyal, Attractive, Kind, And Trustworthy), eight separate 2 × 2 mixed design analysis of variances (ANOVA) were conducted (see Table 2 for descriptive statistics). The between-subjects variable in each ANOVA was Participant Relationship Status (i.e., partnered or single) and the within-subjects variable was Donor Relationship Status (i.e., partnered or single). The family-wise error rate was adjusted for the eight comparisons made such that the alpha-level was set at 0.006 (i.e., 0.05/8).

**Table 2:** Single and partnered women’s ratings of single and partnered men’s faces.

	Entire sample (Women) (*n* = 82)		Partnered women (*n* = 40)		Single women (*n* = 42)	
	Donor relationship status (Male)		Donor relationship status (Male)		Donor relationship status (Male)	
Face ratings	Single	Partnered	Single	Partnered	Single	Partnered

	Mean (SD)	Mean (SD)	Mean (SD)	Mean (SD)	Mean (SD)	Mean (SD)
Masculine	3.47 (1.25)	2.83 (1.05)	3.76 (1.27)	2.67 (1.07)	3.17 (1.17)	2.99 (1.02)
Good partner	1.71 (1.18)	1.80 (1.23)	1.59 (1.24)	1.50 (1.16)	1.83 (1.11)	2.10 (1.02)
Sexy	2.11 (1.34)	1.80 (1.08)	2.18 (1.36)	1.72 (0.99)	2.04 (1.33)	1.89 (1.16)
Intelligent	3.83 (0.80)	3.77 (0.81)	3.84 (0.77)	3.92 (0.74)	3.83 (0.84)	3.62 (0.87)
Loyal	2.94 (1.07)	3.18 (1.02)	2.91 (0.93)	3.44 (0.56)	2.97 (1.20)	2.94 (1.28)
Attractive	2.42 (1.27)	2.28 (1.15)	2.41 (1.35)	2.23 (1.12)	2.43 (1.19)	2.34 (1.89)
Kind	3.31 (1.03)	3.79 (0.80)	3.05 (1.10)	3.92 (0.67)	3.57 (0.89)	3.67 (0.90)
Trustworthy	3.15 (1.02)	3.41 (0.86)	3.02 (1.10)	3.59 (0.80)	3.27 (0.91)	3.22 (0.88)

**FIGURE 1:**
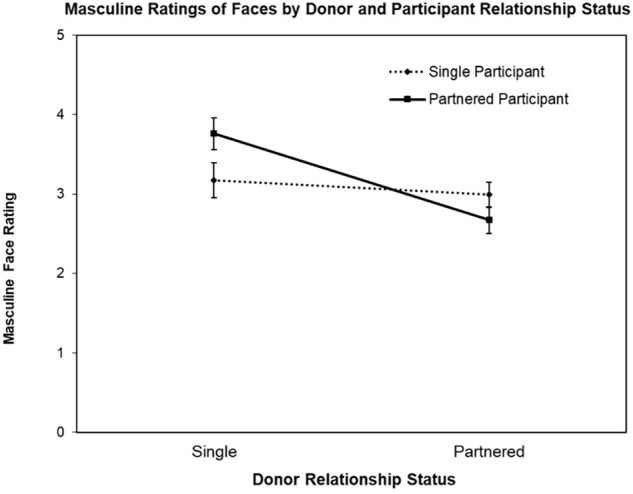
Mean Masculine ratings (±SE) of donor faces (single vs. partnered) by participant relationship status (single vs. partnered).

The first ANOVA was conducted with the face Masculine ratings as the DV, revealing a significant main effect for Donor Relationship Status, *F*(1,77) = 18.76, *p* < 0.001, $\eta_{p}^{2}$ = 0.20 and interaction for Donor Relationship Status by Participant Relationship Status, *F*(1,77) = 9.70, *p* = 0.003, $\eta_{p}^{2}$ = 0.11 (see Figure 1). The main effect for Participant Relationship Status was not significant (*F* < 1). Follow-up contrast testing revealed the nature of the interaction, that is, while partnered female participants rated single men’s faces as more masculine than partnered men’s faces, *t*(39) = 5.72, *p* < 0.001, *d’* = 0.93, single women did not rate partnered and single men’s faces differently on Masculine, *t* < 1.

**FIGURE 2:**
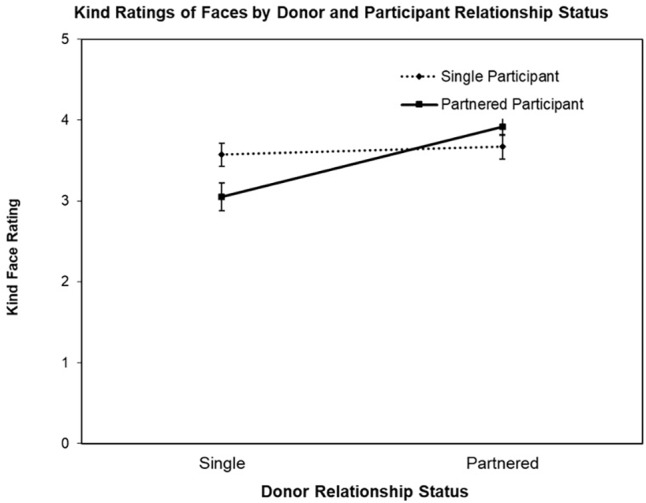
Mean Kind ratings (±SE) of donor faces (single vs. partnered) by participant relationship status (single vs. partnered).

The second ANOVA was conducted with the face Kind ratings as the DV, revealing a significant main effect for Donor Relationship Status, *F*(1,77) = 14.95, *p* < 0.003, $\eta_{p}^{2}$ = 0.16 and interaction for Donor Relationship Status by Participant Relationship Status, *F*(1,77) = 9.53, *p* = 0.003, $\eta_{p}^{2}$ = 0.11 (see Figure 2). The main effect for Participant Relationship Status was not significant (*F* < 1). Follow-up contrast testing revealed the nature of the interaction, that is, while partnered female participants rated partnered men’s faces as appearing kinder than single men’s faces, *t*(39) = 4.94, *p* < 0.001, *d’* = 0.95, single women did not rate partnered and single men’s faces differently on Kind, *t* < 1.

The third ANOVA was conducted with the face Trustworthy ratings as the DV, revealing that the main effect for Donor Relationship Status was not significant, *F*(1,77) = 3.68, *p* = 0.059, $\eta_{p}^{2}$ = 0.0, nor was the main effect for Participant Relationship Status (*F* < 1). While a significant interaction for Donor Relationship Status by Participant Relationship Status was found [*F*(1,77) = 5.45, *p* = 0.022, $\eta_{p}^{2}$ = 0.07], this effect was not significant based on the adjusted alpha-level.

The fourth ANOVA was conducted with the face Loyalty ratings as the DV, revealing that the main effect for Donor Relationship Status was not significant, *F*(1,77) = 3.87, *p* = 0.053, $\eta_{p}^{2}$ = 0.05, nor was the Participant Relationship Status main effect, *F*(1,77) = 1.30, *p* = 0.26, $\eta_{p}^{2}$ = 0.02. While a significant interaction for Donor Relationship Status by Participant Relationship Status was found [*F*(1,77) = 4.59, *p* = 0.035, $\eta_{p}^{2}$ = 0.05], this interaction effect was rendered non-significant based on the adjusted alpha-level.

The next four ANOVAs conducted revealed no significant main or interaction effects (with 8 of 12 *F*-values < 1) indicating that partnered and single women did not rate partnered and single men’s faces different on ratings of good partner, sexy, intelligent, and attractive.

### Exploratory Analyses: Do BO Ratings Predict Face Ratings?

In order to determine whether BO Characteristics ratings predicted Face Characteristics ratings, a Spearman’s rho correlation analysis was conducted, which overall, revealed favorable BO ratings (i.e., Sexy and Like) were associated with favorable face ratings (e.g., Attractive, Intelligent; see Table 3). For example, higher BO Like ratings were significantly correlated with rating faces more Attractive (*r* = 0.29, *p* = 0.008), Masculine (*r* = 0.30, *p* = 0.007), Sexy (*r* = 0.26, *p* = 0.019), and someone who would make a Good Partner (*r* = 0.33, *p* = 0.003). The inter-correlations among the face ratings were positive and statistically significant (except for four); the lowest was between Intelligent and Good Partner (*r* = 0.12, *p* = 0.30) and the highest was between Sexy and Attractive (*r* = 0.89, *p* < 0.001). The inter-correlations among the BO ratings were mostly positive and statistically significant, except for those with the Strong ratings. The lowest significant correlation was between Familiarity and Sexy ratings (*r* = 0.48, *p* < 0.001) and the highest was between Sexy and Like (*r* = 0.78, *p* < 0.001; see Table 3).

**Table 3:** Body odor and face ratings correlations (*N* = 82).

	2.	3.	4.	5.	6.	7.	8.	9.	10.	11.	12.	13.
(1) Sexy BO	0.48^∗∗^	0.78^∗∗^	0.50^∗∗^	−0.02	0.25^∗^	0.33^∗∗^	0.30^∗∗^	0.17	0.06	0.31^∗∗^	0.30^∗∗^	0.08
(2) Familiarity BO		0.52^∗∗^	0.72^∗∗^	0.06	−0.02	0.11	0.06	0.10	−0.05	0.18	−0.04	0.09
(3) Like BO			0.56^∗∗^	−0.10	0.29^∗∗^	0.33^∗∗^	0.24^∗^	0.15	0.06	0.30^∗∗^	0.26^∗^	0
(4) Similarity BO				0.12	0.15	0.34^∗∗^	0.12	0.16	0.08	0.46^∗∗^	0.10	0.10
(5) Strong BO					0.15	0.09	0.16	0.13	0.11	0.17	0.14	0.12
(6) Attractive Face						0.70^∗∗^	0.27^∗^	0.32^∗∗^	0.31^∗∗^	0.48^∗∗^	0.89^∗∗^	0.19
(7) Good Partner Face							0.23^∗^	0.25^∗^	0.23^∗^	0.47^∗∗^	0.68^∗∗^	0.12
(8) Intelligent Face								0.57^∗∗^	0.53^∗∗^	0.22^∗^	0.29^∗∗^	0.55^∗∗^
(9) Kind Face									0.66^∗∗^	0.31^∗∗^	0.32^∗∗^	0.82^∗∗^
(10) Loyal Face										0.28^∗^	0.30^∗∗^	0.71^∗∗^
(11) Masculine Face											0.44^∗∗^	0.20
(12) Sexy Face												0.19
(13) Trustworthy Face												

*Results based on Spearman’s rho correlations. ^∗^p < 0.05, ^∗∗^p < 0.01. BO = body odor.*

The ANOVA results reported above demonstrated that partnered and single participants rated partnered and single donors differently, specifically on the BO Strong ratings and a subset of the face ratings (i.e., Masculine, Loyal, Kind, and Trustworthy). Therefore, we explored the correlations amongst the ratings indicated by the ANOVA findings to determine the nature of the differences between partnered and single females’ ratings. This exploration revealed that the largest discrepancies were all based on ratings of partnered donors’ BO and faces. The largest discrepancy was the correlation between BO Strong and Face Trustworthy ratings: specifically, for the ratings given by partnered women, we found a negative correlation (*r* = -0.35, *p* = 0.025) whereas for the ratings given by single women, we found a positive correlation (*r* = 0.11, *p* = 0.51). A Fisher’s r-to-z transformation comparison test indicated these two correlations were significantly different (*Z* = 2.07), confirming that higher BO Strong ratings were associated with lower Face Trustworthy ratings for partnered women but no such relationship existed for single women. While there were other similarly large discrepancies between partnered and single participants’ ratings, none were significantly different.

## Discussion

Consistent with our hypothesis, single men’s BO was rated as smelling stronger than the BO of partnered men. We also found that single men’s faces were rated as more masculine than partnered men’s faces, but only among partnered women. Moreover, partnered women rated partnered men’s faces as kinder, more trustworthy and loyal than single men’s faces, but single females rated partnered and single men’s faces similarly on these characteristics. Finally, the results showed favorable BO ratings were correlated with favorable ratings of the corresponding faces. Although testosterone levels were not directly tested here, the current study’s findings are congruent with previous research showing that single and partnered males can be differentiated based on their testosterone levels (e.g., Van Anders and Goldey, 2010), that higher testosterone levels are associated with a stronger smelling BO (Rantala et al., 2006) and that more intense BOs are rated more masculine smelling (Havlíček and Lenochova, 2006).

An obvious question is; why would a single male’s BO smell different from that of a partnered man’s BO? The social neuroendocrinology theoretical framework (van Anders and Watson, 2006) helps frame a possible answer to this question. Specifically, BOs are the manifestation of our current endocrinology (e.g., low or high testosterone levels) which signal the fitness, viability, and/or availability of a potential mate. Based on their study’s results, Van Anders and Goldey (2010) concluded that single males have higher levels of testosterone than partnered males because of the sexual competition associated with being single and that low testosterone levels are associated with bond maintenance. From an evolutionary perspective, it may be advantageous for women to be able to detect the chemosignals that connote coupledom and ultimately avoid courting partnered males (especially with offspring) due to the relatively reduced resources they can offer.

An alternative explanation is that single men’s BO may smell more intense than partnered men’s BO because of their poorer health and/or hygiene. Evidence for this assertion comes from research showing single men have poorer physical and mental health outcomes than partnered men (Hu and Goldman, 1990) which may manifest as poorer hygiene and therefore BO. Further evidence comes from research showing married men are significantly more likely to seek health care due to their wives’ influence compared to unmarried men (Norcross et al., 1996). While we found no evidence that single men were less healthy than partnered men based on the fact there were no significant group differences in terms of BMI, the positive health impact of having a partner may explain our findings.

The current study’s finding that single men’s faces were rated significantly more masculine than partnered men’s faces (among partnered women only) is consistent with previous research showing higher testosterone levels are associated with more intense smelling BO (Rantala et al., 2006); especially when considered in conjunction with the finding that single men have higher levels of testosterone than partnered men (e.g., Van Anders and Goldey, 2010). Given higher testosterone levels are associated with more masculine facial features (Penton-Voak and Chen, 2004), it is possible single males in the current sample had higher levels of testosterone. However, a single man’s facial features are unlikely to change overnight unlike their relationship status, so alternative explanations for the differences in Masculine ratings for partnered and single men’s faces must be considered. While facial features do change with age, partnered males were not older than single males so age can be ruled out as an explanation for group differences in facial masculinity. Having a beard was also excluded as an explanation for higher Masculine ratings of single men’s faces but it remains possible that individual differences in what constitutes a “masculine” face may, to some extent, account for the finding.

While it is curious that only partnered women rated single men’s faces as more Masculine than partnered men’s faces, previous research indicates partnered women in the fertile phase of their menstrual cycle (compared to those in their non-fertile phase) find single men’s faces more attractive than partnered men’s faces, especially if they are masculine-versus feminine-looking (Bressan and Stranieri, 2008). A limitation of the current study was that participants’ menstrual cycles were not assessed so we cannot conclude whether menstrual cycle phase influenced their face masculinity ratings. Further limitations include not supplying donors with non-perfumed body cleansing products or specifying a specific duration of exercise, which may have contributed to variability in the quality and nature of the stimuli collected.

The correlations between BO and face ratings revealed a consistent pattern of results indicating favorable BO ratings were associated with favorable face ratings. While the current study’s findings are congruent with previous findings, the positive relationship between BO and face ratings has largely been demonstrated with female participants in the fertile phase of their menstrual cycle (Rikowski and Grammer, 1999; Thornhill and Gangestad, 1999). However, the correlation between BO like ratings and face attractiveness ratings for low fertile compared to high fertile women in both studies (i.e., Rikowski and Grammer, 1999; Thornhill and Gangestad, 1999) were not significantly different, suggesting no reliable group differences. Moreover, Allen et al. (2016) found women’s ratings of masculinity for men’s BO was positively and significantly correlated with face masculinity ratings, although the women’s menstrual phase was not recorded in their study, either. As we could not compare the BO and face rating correlations based on a participant’s menstrual phase, it remains possible that differences exist between the low and high fertility phases of the menstrual cycle.

While the current results show a single man’s BO smells more intense and their face appears more masculine than a partnered man’s, the findings are preliminary and require replication. A specific aim of future research would be to determine whether testosterone levels are responsible for the differences in BO and face ratings between single and partnered men found in the current study. This could be achieved in a single study using the same participants with the aim to (a) replicate the finding that single men’s BO smells more intense than partnered men’s BO; (b) replicate the finding that single men’s faces are rated more masculine than partnered men’s faces; (c) confirm that single men have higher levels of testosterone than partnered men; (d) assess women’s menstrual cycle phase, and (e) comparing an individual’s BO while single and coupled. Future studies would also benefit from ruling out alternative explanations for BO differences between single and partnered men, such as those associated with poor physical and mental health.

## Author Contributions

MM and RS was involved in the study design, data and analyses, and production and editing of the final document.

## Conflict of Interest Statement

The authors declare that the research was conducted in the absence of any commercial or financial relationships that could be construed as a potential conflict of interest. The handling Editor declared a past co-authorship with one of the authors RS.
